# Noise destroys feedback enhanced figure-ground segmentation but not feedforward figure-ground segmentation

**DOI:** 10.3389/fphys.2012.00274

**Published:** 2012-07-17

**Authors:** August Romeo, Marina Arall, Hans Supèr

**Affiliations:** ^1^Faculty of Psychology, Department of Basic Psychology, Universitat de BarcelonaBarcelona, Spain; ^2^Institute for Brain, Cognition and Behavior (IR3C)Barcelona, Spain; ^3^Catalan Institution for Research and Advanced Studies (ICREA)Barcelona, Spain

**Keywords:** computer model, contextual modulation, cortical state, figure-ground, primary visual cortex, spiking neurons, v1, visual perception

## Abstract

Figure-ground (FG) segmentation is the separation of visual information into background and foreground objects. In the visual cortex, FG responses are observed in the late stimulus response period, when neurons fire in tonic mode, and are accompanied by a switch in cortical state. When such a switch does not occur, FG segmentation fails. Currently, it is not known what happens in the brain on such occasions. A biologically plausible feedforward spiking neuron model was previously devised that performed FG segmentation successfully. After incorporating feedback the FG signal was enhanced, which was accompanied by a change in spiking regime. In a feedforward model neurons respond in a bursting mode whereas in the feedback model neurons fired in tonic mode. It is known that bursts can overcome noise, while tonic firing appears to be much more sensitive to noise. In the present study, we try to elucidate how the presence of noise can impair FG segmentation, and to what extent the feedforward and feedback pathways can overcome noise. We show that noise specifically destroys the feedback enhanced FG segmentation and leaves the feedforward FG segmentation largely intact. Our results predict that noise produces failure in FG perception.

The task known as Figure-ground (FG) segmentation is the assignment of visual elements to either objects or background, and constitutes a primary step in visual perception. In the brain, visual features are detected by neurons by means of their feedforward defined classical receptive field, whereas contextual influences beyond the classical receptive field have been interpreted as the neural substrate of FG segmentation. In the primary visual cortex (V1), feedback projections covering large parts transmit extra-classical receptive field information (Angelucci et al., 2002), and are considered to be critical for FG segmentation (Lamme and Roelfsema, 2000). This assumption has been developed in many theoretical and computational models (Sporns et al., 1991; Sun et al., 1999; De Kamps and van der Velde, 2001; Grossberg and Williamson, 2001; Wersing et al., 2001; Roelfsema et al., 2002; Thielscher and Neumann, 2003; Deco and Lee, 2004; Baek and Sajda, 2005; Zhaoping, 2005; Bhatt et al., 2007; Craft et al., 2007; Jehee et al., 2007; Zwickel et al., 2007; Domijan and Setić, 2008; Wagatsuma et al., 2008) that explain FG segmentation by recurrent processing through horizontal and/or feedback connections.

Spike bursts are evoked by feedforward stimulus input whereas cortical feedback modulates the stimulus evoked activity. The role of feedback in not clear but it is believed that feedback transmits top-down attention signals (Theeuwes, 2010). The findings are in agreement with the results of a recent computational modeling study showing that feedback enhances FG segmentation (Supèr and Romeo, 2010, 2011; Supèr et al., 2010, see also Supèr and Lamme, 2007a). This enhancement was seen to occur by a change in cortical state (Le Masson et al., 2002; Supèr and Lamme, 2007a), i.e., a change in the firing pattern from a bursting into a tonic mode (Sherman, 2001). In monkey visual cortex a switch in cortical state also has been observed during FG segmentation (Supèr et al., 2003b; van der Togt et al., 2006). Thus, both cortical state and cortical feedback are crucial for conscious FG perception (Pascual-Leone and Walsh, 2001; Supèr and Lamme, 2007a) and it is, therefore, conceivable that FG segmentation by feedback operates by controlling sensory-evoked spiking pattern. For instance, Sillito et al. (2006) manipulated feedback from cortical layer 6 to the thalamus *in vivo* by focally injecting a GABAb receptor antagonist into the cortex. As a consequence of this manipulation, two-thirds of the studied thalamic relay cells changed their firing patterns. Some relay cells showed a shift from bursting to more tonic firing. So his experiment shows that changes in the strength of corticothalamic feedback can cause shifts in burst probability of thalamic relay cells. Further evidence of corticothalamic control of relay cell bursting comes from the rat somatosensory cortex (Fanselow et al., 2001; Wiest and Nicolelis, 2003).

Sometimes FG segmentation fails (Supèr et al., 2003b; van der Togt et al., 2006) and currently it is not known what causes this failure. FG activity follows the initial burst response to the visual stimulus and forms part of the late tonic stimulus response. Bursts are believed to be less affected by noise (Cecchi et al., 2000; Du et al., 2010) and are important to overcome the synaptic transmission failure. Noise can make the burst durations of periodic regimes, however, variable (Rowat and Elson, 2004). On the contrary, according to results from computational modeling studies tonic firing mode appears to be much more sensitive to noise (Finke et al., 2008) and sufficient noise can convert tonic firing into bursting (Rowat and Elson, 2004) possibly by affecting the interspike interval (Rowat and Elson, 2004; Du et al., 2010). Thus noise may have different effects on the feedforward and on the feedback contributions to FG segmentation. In the present work, we try to determine whether the presence of Gaussian noise can impair FG segmentation, and to what extent the feedforward and feedback pathways can overcome noise. We show that noise specifically destroys the feedback enhanced FG segmentation and leaves the feedforward FG segmentation largely intact. The results of our study predict that noise produces failure in FG perception.

## Materials and methods

### Model architecture

**Figure 1:**
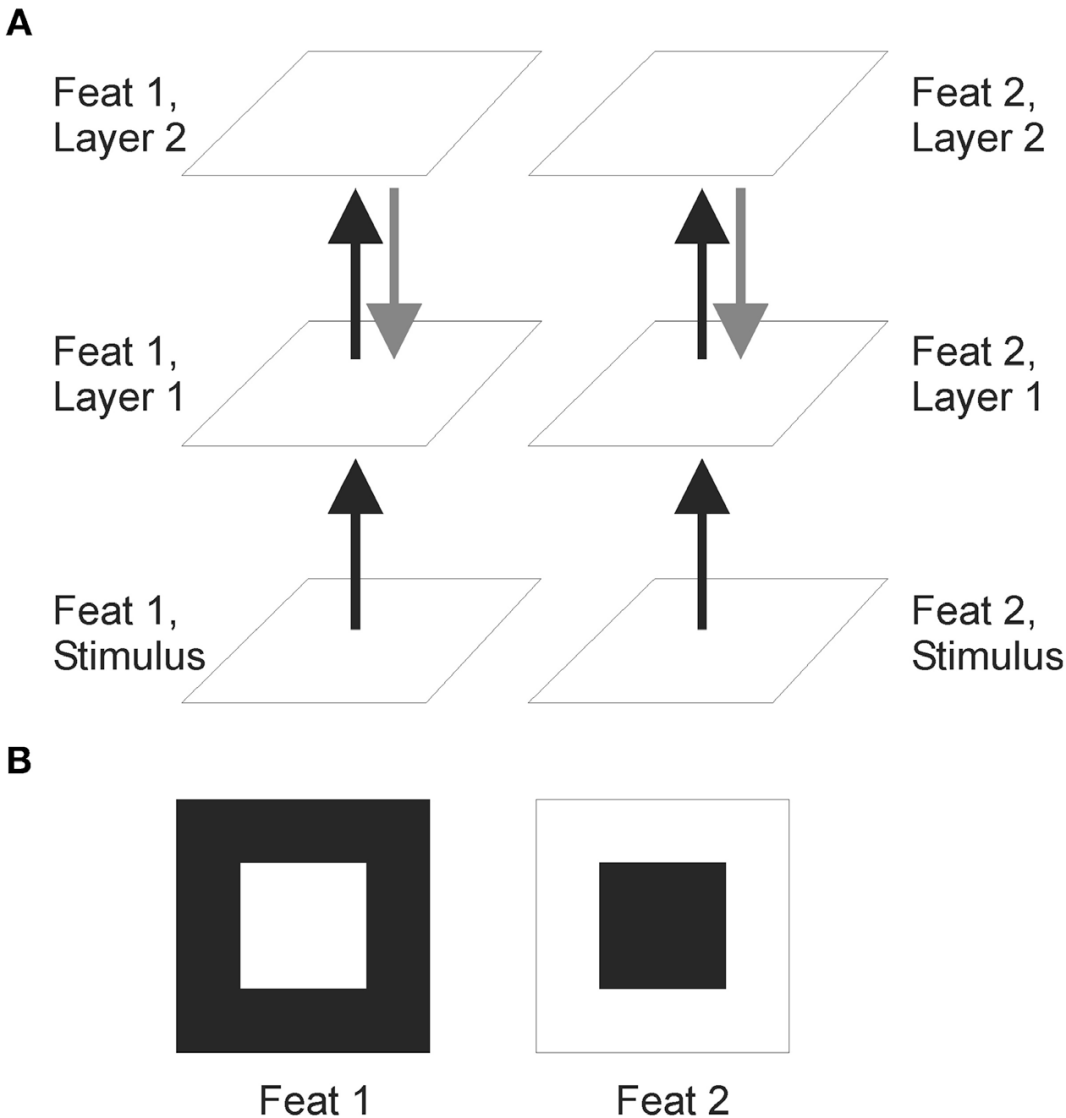
**Schematic representation of the model. (A)** Network made of two layers including two features in separate channels. Black lines indicate feedforward pathways and gray lines show optional feedback pathways. **(B)** The two input features, sometimes referred to as “feat 1” and “feat 2.” In a successful FG segmentation, both spike maps on layer two should signal the figure—and not the background—, i.e., both of them have to look like “feat 1” itself.

The model consists of stimuli representations and two layers, each containing two separate arrays of *N* × *N* neurons of the Izhikevich type (Izhikevich, 2003; see Figure 1). Most of times we use *N* = 64. The two arrays of each layer represent two neuronal cell populations with opposite preference for a given feature. Of course, topological properties of the brain are much more complex than the picture offered by the presented model. For instance, the relationship between structure and function has been illustrated in (Bock et al., 2011), showing the existence of a large number of convergent inputs onto inhibitory neurons (although the result might depend on the size of the analyzed sample). The existence of “hubs” is related to small-world networks and scale-free networks (for a review see Sporns et al., 2005). For a detailed quantitative map of the circuitry of primary visual cortex (see e.g., Binzegger et al., 2004). Nevertheless each layer is ascribed to a visual region. Neurons in the first layer transform continuous or graded input into spike activity and may represent the retina, which provide reliable input to the cortex. The second layer can be regarded as V1 where neural correlates of FG segmentation are observed (Lamme, 1995; Supèr et al., 2001).

### Connections

Feedforward connections between layers have, in general, excitatory and inhibitory contributions. All excitatory connections are retinotopic (point-to-point connections) where the neuron at site (*j, k*) in one layer solely connects to the neuron at site (*j, k*) in the next layer. Thus the excitatory part of a neuron's receptive field has size one. The pattern of inhibitory connections differs between layers. Neurons in the first layer do not receive inhibitory signals from the FG input, although they will optionally receive inhibition from layer 2, i.e., feedback. In the second layer all neurons of a feature map receive inhibition from all neurons located in the same feature map of the first layer, in agreement with observations of large numbers of convergent inputs onto inhibitory neurons in the cortex. Inhibition is achieved by assigning negative weights to the connections. No intra-laminar connections, i.e., horizontal connections between neurons within or across feature maps, are included in the network architecture.

### Inputs

The studied figures are arrays of *N* × *N* pixels containing a centered square. Input arrays are binary (0 or 1) and correspond to the preference of a single visual feature, like luminance, orientation, direction of motion, color etc. In other words, 1 stands for optimal tuning whereas 0 is the opposite. For every shape we include its binary complementary, which represents the reverse preference of the visual feature. These two arrays are referred to as feature map 1 and feature map 2, or “feat 1” and “feat 2.” Together they form the “figure-ground texture.”

### Neuronal cell type

We opted to use the spiking neurons of Izhikevich (2003). These neurons combine the biological plausibility of Hodgkin–Huxley-type dynamics and the computational speed of integrate-and-fire neurons, and can produce a wide variety of firing patterns exhibited by real biological neurons. We choose our neurons to be phasic bursting, which report the beginning of their activity by transmitting a burst.

### Model dynamics

Cell dynamics is described by the “simple” spiking model of Izhikevich (Wiest and Nicolelis, 2003), defined by the system of differential equations

(1) $$\begin{array}{c} \text{C}\frac{dV}{dt}=\alpha V^{2}+\beta V+\gamma -u+I\\ \begin{array}{c} \frac{du}{dt}=a\left(bV-u\right), \end{array} \end{array}$$

and the after-spike reset rule

(2) $$\begin{array}{c} \text{if }V\geq V_{\text{sp}}\text{, then }\{\begin{array}{c} V\leftarrow c\\ u\leftarrow u+d. \end{array} \end{array}$$

The *C*, *V*, *u*, *I*, and *t* symbols indicate membrane capacitance, membrane potential, recovery variable, input intensity and time, respectively. Expectable potential variations are of the order of “a few” mV per ms. Therefore, admitting that typical intensities are of “a few” μA, capacitances should be of the order of μF and for definiteness we set *C* = 1 μF. The spike limit is set at *V*_sp_ = 30 mV. As for the other parameters, *a* is a time scale for the *u* evolution, *b* measures the recovery sensitivity, *c* is the reset value for *V*, and *d* is the height of the reset jump for *u*. We adopt *a* = 0.02 (ms)^−1^, *b* = 0.25 μA/mV, *c* = −55 mV, *d* = 0.05 μA, which correspond to the phasic bursting type of the Izhikevich neuron (Izhikevich, 2003), and the α, β, γ symbols indicate fitted constants, with the values α = 0.04 μA/(mV)^2^, β = 5 μA/(mV), γ = 140 μA. The initial conditions at *t*_0_ (to us *t*_0_ = 0) are

(3) $$\begin{array}{c} V(t_{0})=c\text{, }u(t_{0})=bV(t_{0}) \end{array}$$

When going from the description of a single neuron to systems of two-dimensional arrays, Eqs. (1–3) are understood for *V* → *V*_*jk*_, *u* → *u*_*jk*_, *I* → *I*_*jk*_, 1 ≤ *j*, *k* ≤ *N*, and these *V*_*jk*_, *u*_*jk*_, *I*_*jk*_ are regarded as coefficients of *N* × *N* matrices, say *V*, *u*, *I*. Numerical integration is performed using the Euler method with a time step Δt = 0.20 ms. In most of our simulations *t* ranges from 0 to *t*_max_ = 100 ms. The aforementioned input matrix *I* is, in general, the result of adding two possible contributions:

(4) $$\begin{array}{c} I_{jk}=I_{ejk}+I_{ijk} \end{array}$$

where *e* stands for “excitatory” and *i* for “inhibitory.” In particular, for layer 1,

(5) $$\begin{array}{c} I_{e}=w_{e}T\\ I_{i}=w_{i}\bar{S_{2}}1\text{ for }t\geq t_{1}+t_{d,}\text{ and 0 otherwise}\\ \begin{array}{c} \bar{S_{L}}=\frac{1}{N^{2}}\sum_{j,k}S_{Ljk} \end{array} \end{array}$$

where *T* is the stimulus itself taken as a matrix, *t*_1_ is the time of the first spike and *t*_*d*_ indicates a chosen delay for the inhibitory action. Thus, inhibition is active only *t*_*d*_ ms after *t* = *t*_1_. *S*_*L*_ is the binary array defined by the presence of spikes on layer *L* [i.e., with ones where condition (2) is satisfied and zeros elsewhere], also called “spike map,” and $\bar{S_{L}}$ indicates the spatially averaged spike map for layer *L*, or “mean *S*_*L*_” Obviously, the $\bar{S_{L}}$ value amounts to the ratio between spiking area and total area. Thus, the inhibitory contribution to layer 1 is a feedback term coming from the spike map of layer *L* = 2. The **1** symbol denotes an *N* × *N* matrix which contains just ones, indicating that the inhibition is spatially constant.

Concerning the inputs to layer 2, they are given by

(6) $$\begin{array}{c} I_{e}=w_{e}S_{1}\\ \begin{array}{c} I_{i}=w_{i}\bar{S_{1}}1 \end{array} \end{array}$$

Since excitatory receptive fields have size one, excitatory signals are point-by-point (retinotopic) copies of *S*_1_, multiplied by the corresponding weight. Again, the inhibitory part is spatially constant. The inhibition is now feedforward, and proportional to the spatial average of *S*_1_. Our chosen weights are *w*_*e*_ = 1 for signals from image to layer 1, *w*_*i*_ = −400 for inhibition from layer 2 to layer 1—if present—, *t*_*d*_ = 5 ms, and *w*_*e*_ = 400, *w*_*i*_ = −700 for signals from layer 1 to layer 2 (when not explicitly written it has to be understood that weights or weight variations are given in the employed current intensity units which, in our system, are μA).

### Critical values

The first issue is to obtain a limit to input values which can cause their receiving neurons to spike. As an approximate bound for non-spiking |spiking regimes we shall take *I* < *I*_*b*_|*I* > *I*_*b*,_ being *I*_*b*_ the *I* value limiting the presence of equilibrium states in Izhikevich's model (1), which amounts to $I_{b}=\frac{{\left(\beta -b\right)}^{2}}{4\alpha }-\gamma$, where α, β, γ, *b* are the parameters in Eq. (1). Thus, the *I*_*b*_ value depends on the cell properties, and for our chosen neuron type *I*_*b*_ ≈ 1.02 μA. In view of the involved scales (see below) using this *I*_*b*_ or just *I*_*b*_ = 0 makes little difference.

Synaptic efficiency has been object of interest from several viewpoints (see e.g., Faure et al., 2000; Hardingham et al., 2010; Paprocki and Szczepanski, 2011) and, among other possible approaches, may be related to the presence of noise. Let *w*_*e*,_ *w*_*i*_ indicate the excitatory and inhibitory weights from middle to top layer. As *w*_*i*_ < 0, *w*_*i*_ = −|*w*_*i*_|. Now, considering the connections between middle and top layer in our model (in the feedforward connection and if applicable in the feedback connection), we shall imagine that the total input includes now a stochastic contribution, i.e.,

$$I_{e}+I_{i}\to I_{e}+I_{i}+\xi ,$$

where ξ is a noise matrix of the same size as *I*_*e*_ or *I*_*i*_.

The incorporation of noise from stimulus to layer 1 will be considered below. Among all the known noise types, Gaussian noise is just one of the most widely used and will be employed, for simplicity, in the present work. Nevertheless, it is in general interesting to consider the effects of noise types with power spectra of the form 1/*f*^*n*^, like in (Nozaki et al., 1999).

We denote by *r* the ratio between figure area and total area. For the ground, the analogous ratio is 1 − *r*. In the absence of noise, the input to layer 2 is given by Eqs. (4) and (6). Taking into account that the excitatory part is retinotopic and the inhibitory contribution amounts to *w*_*i*_ multiplied by the ratio spiking area/total area, one realizes that the continuation of spiking inside the figure area requires

$$w_{e}-\left|w_{i}\right|r>I_{b},$$

and, at the same time, to maintain the non-spiking regime inside the ground area calls for

$$w_{e}-\left|w_{i}\right|\left(1-r\right)<I_{b}.$$

The last two relations lead to

$$\frac{w_{e}-I_{b}}{1-r}<\left|w_{i}\right|<\frac{w_{e}-I_{b}}{r}.$$

From these inequalities it follows that necessarily *r* ≤ 1/2, otherwise there can be no solution. Of course, this “critical” ratio is the result of a rather rough estimation. In actual simulations, the process fails for somewhat smaller *r* values (for *N* = 64 this failure is already evident at *r* ≈ 0.4). When the figure size is safely below the critical value, then, for a given excitatory weight there is a limited range of inhibitory weights. The mid-value of this range is ${\left|w_{i}\right|}_{\text{mid}}=\frac{1}{r(1-r)}(w_{e}-I_{b})$ and the “semi-width” or half-range is given by $\Delta \left|w_{i}\right|=\frac{1-2r}{r(1-r)}(w_{e}-I_{b})$. We set figure and total area sides at 32 and 64 pixels, which yield *r* = 1/4. Taking *w*_*e*_ = 400, these formulas give |*w*_*i*_|_mid_ = 1064, Δ|*w*_*i*_| = 532. Thus, for *w*_*i*_ around 1000, Δ|*w*_*i*_| ≈ 500 can be interpreted as a noise amplitude which causes the FG segmentation to go significantly wrong. This “amplitude” is just a typical scale, like the σ value in the case of a Gaussian noise. The described set-up has been simulated for increasing σ's from 0 to values of the order of Δ|*w*_*i*_|. The unwritten units for σ are the same as for current intensities: in our studied case, μA.

### Figure-ground index

The plotted FG efficiency or “modulation index” is a measure defined as:

$$M=\frac{F_{m}-G_{m}}{F_{m}+G_{m}},$$

where *F*_*m*,_ *G*_*m*_ are mean values, including both “features,” of space-averaged spiking rates for sites on figure and ground areas, respectively (Supèr and Romeo, 2012).

## Results

**Figure 2:**
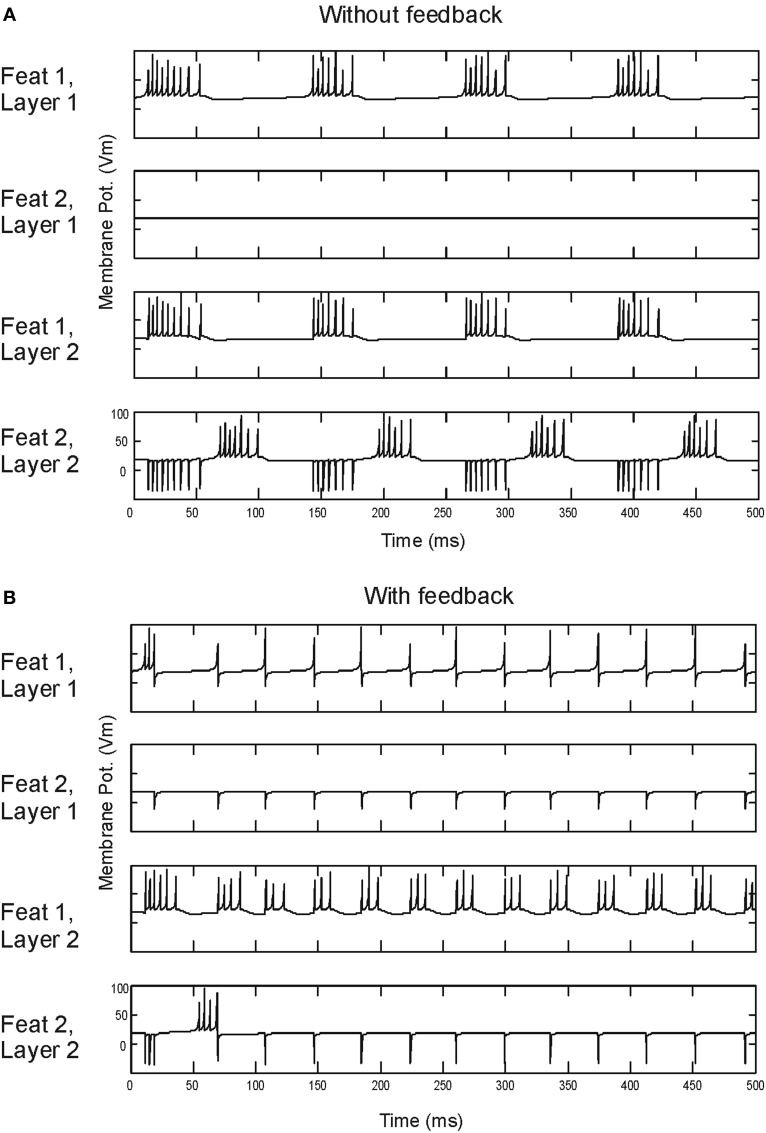
**Spiking responses.** Time evolution of membrane potentials, in mV, at the middle point for each layer and feature map of the model without **(A)** and with **(B)** feedback.

Cells on layer 1 transform stimuli input into spike maps by retinotopic signals (i.e., point-to-point). In the feedforward model, responses take the form of transient bursts (Figure 2A). When feedback is in included spike responses become more tonic (Figure 2B). Cells on layer 2 integrate the information from layer 1 through center-surround “receptive fields” made of excitatory retinotopic centers and inhibitory surround performing a global space average. In the first feature channel “feat 1,” cells on the central figure produce similar spike bursts. For the “feat 2” channel, cells in the central part of layer 1 are quiescent. The relatively large surround area—the background—causes a strong suppression which can neutralize the retinotopic excitation. When this happens, FG segmentation occurs. Thus, FG segmentation is achieved by the model architecture and by a proper balance between excitation and inhibition (see Supèr et al., 2010 for details). Next we go on to consider the role of noise in FG segmentation.

### Figure-ground index and noise

**Figure 3:**
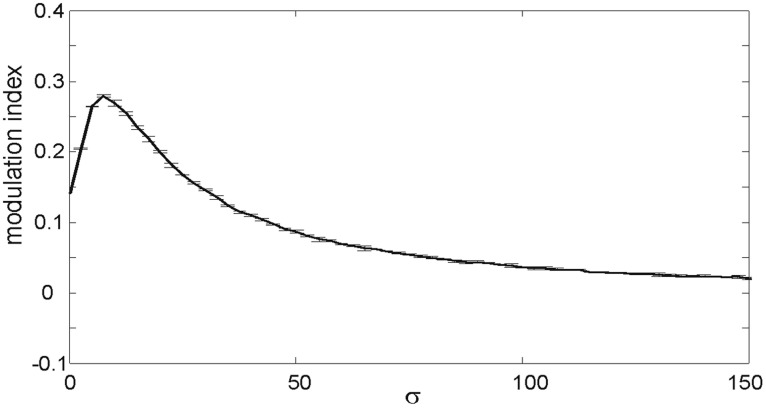
**Strength of figure-ground modulation in a feedforward model.** Modulation indices for simulations with Gaussian white noise of increasing σ (0 ≤ σ ≤ 150). Error bars indicate standard deviations.

Without noise or feedback our model yields a modulation index *M* = 0.14. When including noise the FG modulation index increases. For increasing σ's FG performance grows to a maximum and slowly decreases (Figure 3). When the evolution is dominated by intense noise, the spatial distribution of spikes is highly random to the extent that *F*_*m*_, *G*_*m*_ are almost equal. As σ reaches the predicted Δ|*w*_*i*_|, the “efficiency” is very close to zero.

**Figure 4:**
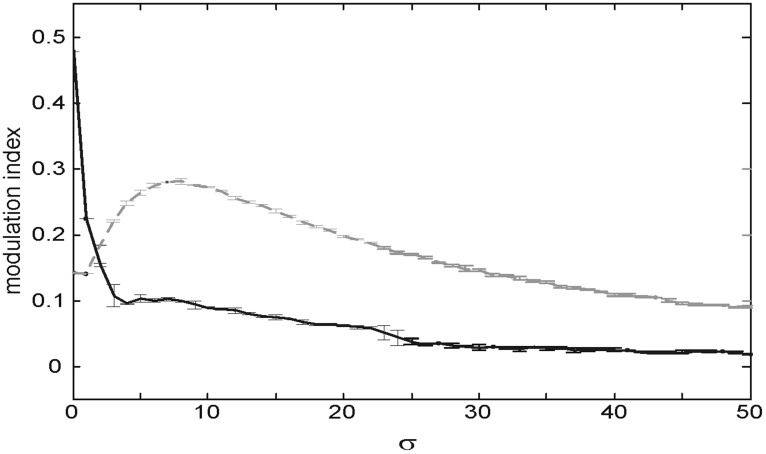
**Strength of figure-ground modulation as a function of noise (σ).** The gray dotted and black lines show FG modulation in feedforward and feedback model, respectively. Error bars indicate standard deviations.

Activating the feedback connections affords a higher FG index of *M* = 0.48, in accordance with previous observations (Supèr and Romeo, 2011). If now a Gaussian noise of increasing σ is added, the FG modulation index decays for σ values ≈ 10 (Figure 4 black trace). Therefore, a noise of this magnitude destroys the enhancing effect of feedback. We interpret the enhancing power of feedback as something measurable by the difference in noise amplitude which yields the same jump in modulation efficiency.

### Space correlations

**Figure 5:**
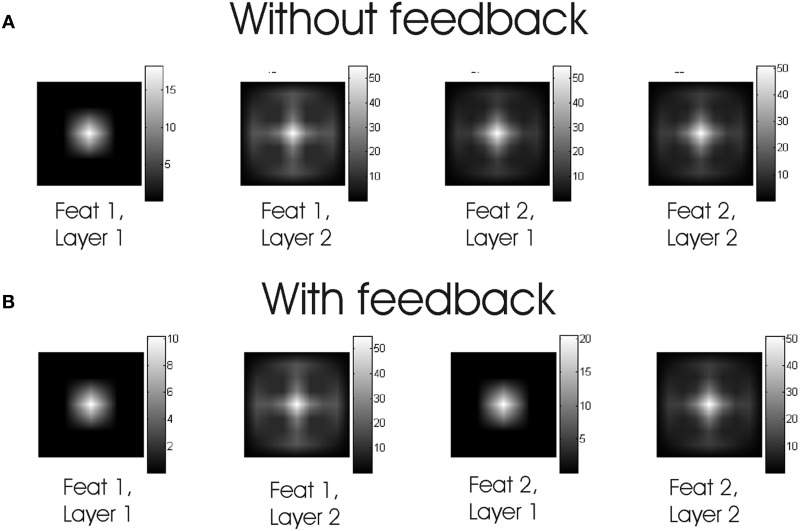
**Time-averaged space correlations without noise.** Model without **(A)** and with **(B)** feedback.

**Figure 6:**
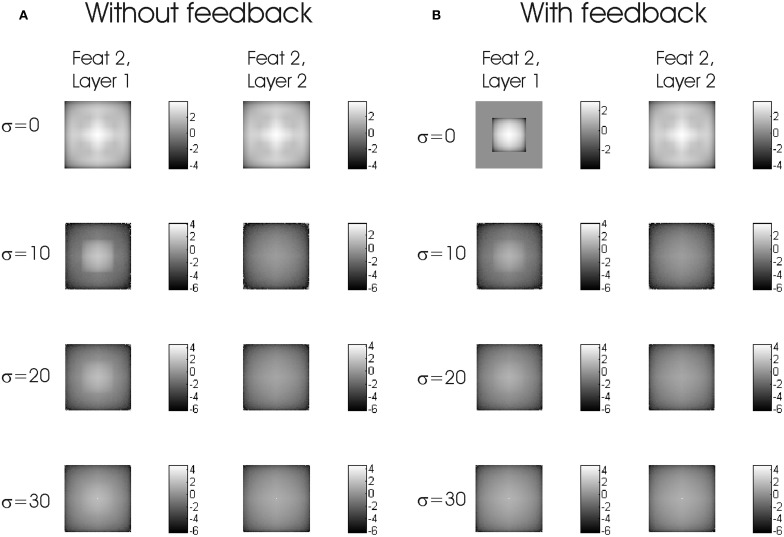
**Logarithms of time-averaged space correlations with Gaussian white noise of increasing σ from 0 to 30. (A)** without feedback. **(B)** with feedback. The part where the correlation is exactly zero has been excluded.

Next we calculated the space correlations. Let C_LF_(*t*) denote the space cross-correlation matrix of the spike map of “feat” *F*, layer *L* with itself at some time *t*, and <C_LF_> the time average of C_LF_(*t*) along a simulation. In the absence of noise (σ = 0), the effect of feedback increases the relative correlation strengths for the figural region of the “feat 1” channel (Figure 5). When noise is added, facilitation in the absence of feedback can be noticed (Figure 6). Feedback gives better results for σ = 0, but from there on there is a monotonic decay. Without feedback, the noiseless case is worse, but there is a relative improvement for noise amplitudes around σ ≈ 10 − 20.

### Noise on both layers

**Figure 7:**
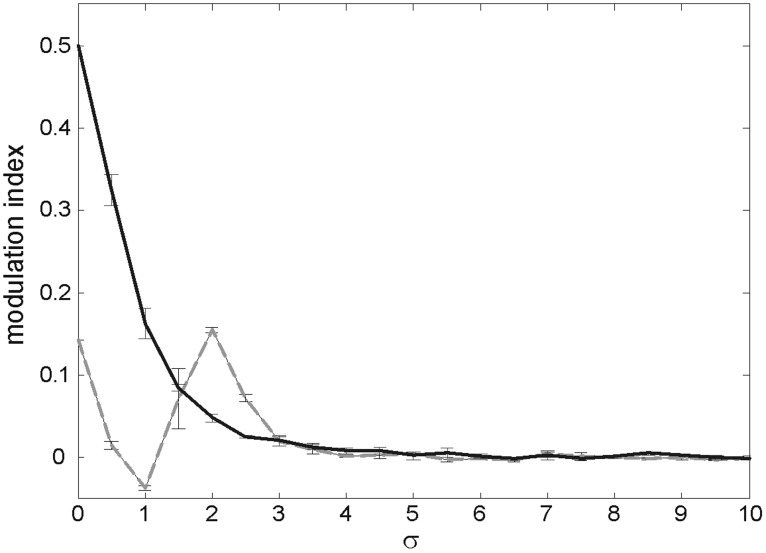
**Modulation index for increasing σ when there is noise in the synapses of layer 1 (in addition to those of layer 2).** Gray trace represents data from model without feedback and black trace with feedback. Error bars indicate standard deviations.

When noise affects synapses on both layers, and not just layer 2 as above, a similar picture emerges. Figure 7 depicts a simulation with noise of the same σ on layer 1 and layer 2. Like the case of noise on layer 2 only, the FG modulation index with feedback is *M* = 0.48 and drops by adding noise. Without feedback the FG index appears to have a minimum before reaching the maximum, and drops to zero with higher σ values.

### Larger network areas

**Figure 8:**
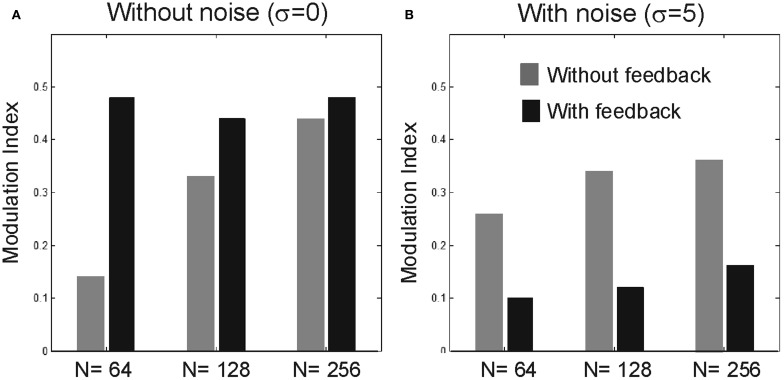
**Effect of larger network sizes.** Bar plots showing the modulation index for sizes *N* = 64, 128, and 256, when σ = 0 **(A)** and σ = 5 **(B)**.

We tested the performance of the model for larger *N* values, namely for *N* = 128 and for *N* = 256 (Figure 8). When σ is zero feedforward FG becomes stronger for larger networks and we observe stronger FG modulation when feedback is included. After including noise (σ = 5), the enhanced FG modulation by feedback has almost vanished (on average 80% decline) while the strength feedforward FG modulation remains similar. Note that unlike the case of *N* = 64, noise-facilitation in the absence of feedback is practically invisible in the larger networks.

### Different figure sizes

**Figure 9:**
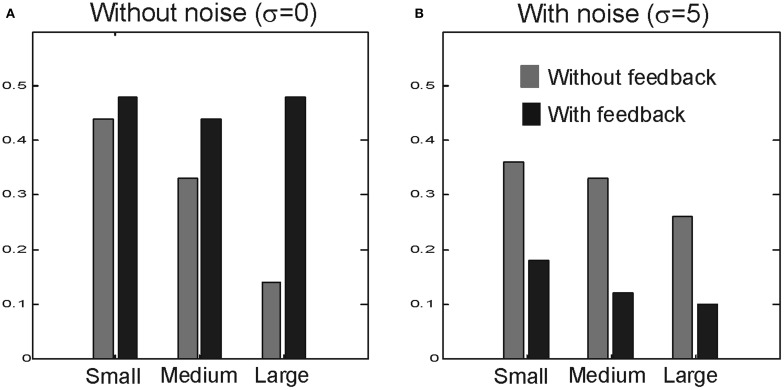
**Effect of figures size.** Bar plots show the modulation index for different figure sizes with *N* = 8 (small), 16 (medium), and 32 (large) when σ = 0 **(A)** and σ = 5 **(B)**.

A change in figure size translates into a change in the balance between excitation and inhibition and, therefore, affects the strength of FG modulation (Supèr et al., 2010) which in turn affects the feedback contribution to FG modulation (Supèr and Romeo, 2011). To further support the idea that noise destroys feedback enhanced FG modulation, we tested the model for smaller figure sizes when the strength of FG modulation becomes stronger. As expected, without feedback FG modulation is stronger for small figure sizes (Figure 9A). Feedback enhanced the FG index. This enhancement is strong when feedforward FG modulation is weak. After including noise (σ = 5), the FG modulation index strongly drops in the recurrent model but not in the feedforward model, even when FG modulation is strong (Figure 9B).

## Discussion

The occurrence and strength of FG modulation to a particular stimulus depends on the state of the visual cortex (Supèr et al., 2003b; van der Togt et al., 2006), and translates into corresponding perceptual performance (Supèr et al., 2001, 2003a, 2007; Supèr and Lamme, 2007b). Cortical state is characterized by the way neurons transmit sensory information, i.e., bursting versus tonic spiking (Bock et al., 2011). In agreement with neurophysiological observations (Le Masson et al., 2002), computer modeling studies (Supèr and Romeo, 2011) demonstrated that this differential gating of feedforward information involves inhibition by feedback projections. In a feedforward scheme neurons performing FG segmentation show a bursting spiking pattern whereas in a recurrent model, i.e., with feedback, neurons show tonic firing patterns. In addition, when feedback is present segmentation of FG is enhanced (Supèr and Romeo, 2011). In this study we show that noise destroys the enhanced FG signal by feedback but not the feedforward FG segmentation.

Sensory information rapidly propagates across the neural system by spike bursts, which are less affected by noise (Cecchi et al., 2000; Du et al., 2010). Our findings are in agreement with these studies as we observe that the strength of FG segmentation is not affected by the appearance of noise when the neurons fire in bursting mode, i.e., in a feedforward connectivity scheme. On the contrary, when neurons respond in a tonic mode we find that noise eliminates the enhancement of the FG signal. This result supports previous evidence demonstrating that noise is a disturbing factor (Faisal et al., 2008), and is in particular harmful during periods of tonic spike responses (Finke et al., 2008).

In a previous paper we showed that segmentation of figure from ground depends on the ratio of FG size, where segmentation occurred for very small as well as for very large figures (Supèr et al., 2010). This finding agrees with human FG perception, where small stimuli are interpreted as figures and larger ones as background, and with the notion that the assignment of figure and ground becomes ambiguous when they have the same size (Barenholtz and Feldman, 2006). Here we show that the feedback enhanced FG modulation occurs for small as well for large figures. However, the enhancement is most noticeable for large figures, when FG modulation is weak. This agrees with our previous study on the role of feedback in FG segmentation (Supèr and Romeo, 2011). In that study we suggest that feedback functions as an attention mechanism. After including noise, the enhanced FG modulation disappears for all figure sizes whereas the feedforward FG modulation remains intact even for small figures when feedforward FG modulation is strong. Thus, noise appears to specifically disrupt feedback FG modulation.

Furthermore, in our study we observe that for relatively small noise levels, noise slightly increases the FG signal in the feedforward model. In contrast, we did not observe any facilitation effect in the feedback model. Such facilitation may occur only when signals are relatively weak and noise can help a sub-threshold value to get over the limit (Faisal et al., 2008). Our observations in the feedforward model support the idea that in spike generating neurons noise can transform threshold nonlinearities by making sub-threshold inputs more likely to cross the threshold, thereby facilitating spike initiation and improving neural-network behavior (Anderson et al., 2000).

To detect a FG stimulus the cortical state has to change during the stimulus presentation period (Supèr et al., 2003b; van der Togt et al., 2006). Previous FG studies predict that this change in cortical state entails a shift from bursting to tonic spiking patterns (van der Togt et al., 2006; Supèr and Romeo, 2011). Disturbing this transition process, noise may prohibit a correct and timely change in neuronal interactions thereby preventing the occurrence of FG perception (see van der Togt et al., 2006 for a discussion). The idea that noise affects the change in spiking mode is in line with studies showing that noise makes burst durations of periodic regimes variable (Rowat and Elson, 2004) and that sufficient noise can convert tonic firing into bursting (Rowat and Elson, 2004). In addition, we speculate that the fluctuation of noise levels may be the cause of the varying strength of FG signals for repeated stimulus (Supèr et al., 2003a, 2007; Supèr and Lamme, 2007b). In conclusion, we speculate that noise turns out to be more destructive for feedback-enhanced FG segmentation than for the purely feedforward process, because of the different spiking regimes.

### Conflict of interest statement

The authors declare that the research was conducted in the absence of any commercial or financial relationships that could be construed as a potential conflict of interest.
